# Implementation support for contingency management: preferences of opioid treatment program leaders and staff

**DOI:** 10.1186/s43058-021-00149-2

**Published:** 2021-04-30

**Authors:** Kelli Scott, Shelly Jarman, Samantha Moul, Cara M. Murphy, Kimberly Yap, Bryan R. Garner, Sara J. Becker

**Affiliations:** 1 Center for Alcohol and Addiction Studies, Brown University School of Public Health, 121 South Main Street, Providence, RI 02903 USA; 2 University of New Haven, 300 Boston Post Road, West Haven, CT 06516 USA; 3 RTI International, 3040 E Cornwallis Rd, Durham, NC 27709 USA

**Keywords:** Contingency management, Implementation support, Opioid use disorder

## Abstract

**Background:**

Contingency management (CM), a behavioral intervention that provides incentives for achieving treatment goals, is an evidence-based adjunct to medication to treat opioid use disorder. Unfortunately, many front-line treatment providers do not utilize CM, likely due to contextual barriers that limit effective training and ongoing support for evidence-based practices. This study applied user-informed approaches to adapt a multi-level implementation strategy called the Science to Service Laboratory (SSL) to support CM implementation.

**Methods:**

Leaders and treatment providers working in community-based opioid treatment programs (OTPs; *N* = 43) completed qualitative interviews inquiring about their preferences for training and support implementation strategies (didactic training, performance feedback, and external facilitation). Our team coded interviews using a reflexive team approach to identify common a priori and emergent themes.

**Results:**

Leaders and providers expressed a preference for brief training that included case examples and research data, along with experiential learning strategies. They reported a desire for performance feedback from internal supervisors, patients, and clinical experts. Providers and leaders had mixed feelings about audio-recording sessions but were open to the use of rating sheets to evaluate CM performance. Finally, participants desired both on-call and regularly scheduled external facilitation to support their continued use of CM.

**Conclusions:**

This study provides an exemplar of a user-informed approach to adapt the SSL implementation support strategies for CM scale-up in community OTPs. Study findings highlight the need for user-informed approaches to training, performance feedback, and facilitation to support sustained CM use in this setting.

Contributions to the literature

- Contingency management (CM) is an evidence-based adjunct to medication for opioid use disorder. Unfortunately, opioid treatment programs (OTPs) face many barriers to implementing CM (e.g., lack of training, resource limitations).
- This study analyzed qualitative data from OTP staff and leaders about their preferences for CM implementation to inform adaptation of a multi-level implementation strategy prior to full-scale testing in a multi-site trial.
- User-informed adaptation of implementation strategies is infrequently applied in the implementation science literature but important for enhancing uptake of both an implementation strategy and the evidence-based practice being implemented.

## Implementation support for contingency management: preferences of opioid treatment program leaders and staff

Opioid use disorder and opioid-related overdose deaths are a major public health crisis in the USA. Medication for opioid use disorder (MOUD) is considered the frontline treatment, typically involving medications including methadone and buprenorphine [1]. Opioid treatment programs (OTPs) are regulated by the Substance Abuse and Mental Health Administration (SAMHSA) and provide medications and counseling to individuals in the community with OUD [2]. Providers working in OTPs face a number of unique challenges including high patient volume and a fast-paced work environment, [3] as well as systems-level issues such as strict federal guidelines regulating patient contact and low reimbursement for services [4]. Combined, these challenges increase the risk of provider burnout and turnover and make it difficult to train and retain the OTP workforce in evidence-based practices [5–7].

Contingency management (CM), or the provision of patient incentives to promote achievement of treatment-related goals, is an evidence-based behavioral intervention with strong support as an adjunctive intervention in OTPs [8]. CM typically involves providing tangible incentives (e.g., gift cards, small prizes) for achieving treatment goals, with positive reinforcement serving to increase the likelihood of continued substance abstinence and/or treatment attendance. Incentives are provided on a consistent (e.g., weekly) schedule, with prizes awarded immediately following achievement of the goal (e.g., a negative urine toxicology screen or attendance at counseling [9]). CM has large effect sizes when delivered in combination with MOUD [8] and has emerged as a superior treatment when compared head-to-head with cognitive behavioral therapy as an adjunctive intervention [10]. Unfortunately, a myriad of barriers have limited OTP providers’ ability to successfully implement CM with fidelity [11, 12].

Traditional approaches to scaling up evidence-based practices like CM often involve didactic workshops, usually as part of continuing education requirements for licensure or for new practice implementation at an agency [12, 13]. Although these trainings are effective for enhancing provider knowledge about evidence-based practices, they are often insufficient for sustaining provider behavior change or new practice implementation [14]. An extensive body of research has shown that didactic training is enhanced when paired with the provision of training and support strategies such as feedback on provider performance and access to an external facilitator [15–17].

The Science-to-Service Laboratory (SSL), developed by the SAMHSA-funded network of Addiction Technology Transfer Centers (ATTCs), is an example of a multi-level implementation strategy that combines three empirically supported elements: didactic training, performance feedback, and external facilitation [18]. These three elements address contextual determinants at both the provider and organizational levels. The SSL typically commences with didactic training, often delivered as a 1-day workshop with experiential learning, followed by ongoing feedback on the fidelity of intervention delivery. These elements address provider-level determinants such as knowledge and perceptions of the intervention. Meanwhile, technology transfer specialists provide external facilitation to address organization-level determinants such as how the intervention fits into the workflow, ensuring time and funding for intervention delivery, provider turnover/retraining, and leadership engagement. The New England ATTC has been evaluating the SSL strategy since 2008 and has described the strategy extensively in prior work (see [18–20]).

In early SSL work, we found that agencies providing substance use treatment were more likely to adopt CM when they participated in all of these SSL elements than when they only completed some of the elements [18]. In a more recent study specifically focused on OTPs, we compared implementation outcomes in seven OTPs that received the SSL to 11 OTPs that only received didactic training-as-usual (without performance feedback or external facilitation). We found that the SSL resulted in both significantly higher adoption and faster implementation of CM when compared to didactic training-as-usual, providing evidence for the additional utility of the performance feedback and facilitation strategies [20, 21].

We employed a user-informed approach grounded within formative qualitative research to understand the unique needs of OTP staff with regard to training and support strategies for CM implementation. Leaders and providers from multiple OTPs shared their preferences regarding CM didactic training, performance feedback, and external facilitation to inform format, content, frequency, and delivery. The long-term goal of this study was to inform the final design of the SSL implementation strategy used in a cluster randomized implementation trial with 30 OTPs throughout New England.

## Methods

### Recruitment

At the time of this study (see [22]), there were 13 OTPs in the state of Rhode Island, all of which were invited to nominate staff for participation. Research staff contacted executive leaders and directors from the OTPs via both phone and email to describe the qualitative study, to describe researcher interest in receiving input from OTPs on the SSL strategy, and to request nominations of eligible staff. Each OTP nominated two providers and two leaders to participate. Eligibility criteria for leaders included supervising or managing providers and at least 6 months of employment at their site. Providers needed to have been employed for at least 3 months and have an active caseload that involved providing psychosocial counseling services to patients at their treatment facility.

### Participant enrollment

The Institutional Review Board at the Miriam Hospital, an affiliate of the Alpert Medical School of Brown University, approved this study and granted a waiver of documented consent (Project Number: 210718 45CFR 46.110(7)): all nominated participants completed informed consent verbally over the phone or in-person at their OTP office. Leaders and providers were invited to participate in 45 to 60-min audio-recorded interviews as part of the informed consent process. Participants were assured of their rights to confidentiality and privacy. Participants were also assured that their responses would not be shared with their employer and no identifying information was included in the data collection. Participants were told that decisions about participation would not be shared with OTP leaders and would not affect their employment at their OTP. Participants were offered $100 for completion of the interview.

### Interview procedures

We conducted audio-recorded interviews both in person and over the phone with providers and leaders. Four interviewers trained by the study PI (SJB) in semi-structured interview methods conducted the interviews one-on-one with participants either on-site at the OTPs or via phone. Interviewers included two postdoctoral fellows (KS and CM), one Bachelor’s level Research Assistant, and one Master’s level Research Assistant. Interviews included questions about a wide range of CM design and training preferences (see Becker et al., 2019 for the interview guide [22]). All providers were given a working definition of CM at the start of the interview to ensure that all participants had sufficient knowledge of CM principles.

The current study focused on questions regarding participants’ preferences for the SSL implementation strategy to scale up CM, including providers’ and leaders’ preferences for didactic training elements (e.g., content, format, and delivery), performance feedback (e.g., how often, by whom), and external facilitation (e.g., how often, how accessed, focal topics). These questions were prioritized to inform our adaptation of the SSL using a user-informed formative evaluation approach [23]. Interviewers also took notes regarding provider demographic characteristics and any feedback received on the interview questions.

### Data analysis

Interview recordings were transcribed and cleaned to ensure the removal of all identifying information. Transcripts were not returned to participants for correction. Three independent coders completed transcript coding and thematic analysis using a reflexive team analysis approach [24, 25]. Coders included two Research Assistants (KY and SM; One Bachelor’s and one Master’s level, both new to the study at the time of coding) and one Postdoctoral Fellow (KS; also an interviewer). The coders collaboratively developed a coding dictionary that included both a priori (i.e., didactic training, performance feedback, and facilitation themes) and emergent themes based on review of the transcripts. The coders then imported all codes and transcripts into NVivo version 12 coding software and applied the dictionary to all 43 transcripts. Two coders each independently coded half the transcripts each, with the third coder coding 20% of the transcripts to ensure inter-rater reliability. Coders met weekly to discuss and resolve coding discrepancies and achieve 100% consensus in coding decisions. Coders also discussed additional emergent themes identified during the coding process and made modifications to the coding dictionary to achieve saturation of themes identified.

After coding was complete, the coding team ran queries in NVivo to identify the most commonly endorsed preferences for CM training format, performance feedback, and external facilitation. The most common themes and sub-themes were tabulated through transcript frequency counts. Exemplar quotes were identified for each theme. Findings were shared with leadership at each OTP to give them the opportunity to provide feedback on the interpretation of results.

## Results

The primary goal of this analysis was to evaluate how front-line providers and organization leadership would adapt a CM-focused implementation strategy at their site, considering their unique organizational context. Table 1 presents characteristics of the final sample, and Table 2 presents a summary of emergent themes and illustrative quotes.

**Table 1:** Participant sociodemographic characteristics (*N* = 43)

	Leaders (***N*** = 21)	Providers (***N*** = 22)	Overall (***N*** = 43)
**Age (years; M(SD))**	42.7 (15.0)	37.9 (13.8)	40.2 (14.4)
**Biological sex**			
Female	14 (67%)	17 (77%)	31 (72%)
Male	7 (33%)	5 (23%)	12 (28%)
**Race**			
White	20 (95%)	20 (91%)	40 (93%)
Other	1 (5%)	2 (9%)	3 (7%)
**Education**			
High school diploma, GED, or bachelor’s degree	12 (57%)	17 (77%)	29 (67%)
Some graduate school or higher	9 (43%)	5 (23%)	14 (33%)
**Experience at OTP (years; M(SD))**	5.8 (8.6)	3.9 (3.5)	4.8 (6.5)

**Table 2:** Themes related to the design of the implementation strategy with definitions and illustrative quotes

Theme	Elements explored	Illustrative quotes
Didactic training	• Training format	Leader: “Usually our training would probably go through a staff meeting ‘cause that’s the best way to get all of us in the same spot ‘cause it’s a designated time off.” Staff: “It would probably be best if we all had one training for the entire site. That way, we’re all getting the same exact information. Oftentimes, with a site as small as us, we end up having multiple—we might all go to the same training but on multiple different days throughout the whole year just because we can’t afford to send everybody all at once.”
	• Training content	Leader: “maybe if there is any type of existing program that works really well, having someone like that, the leader of that come over here and talk to us about it” Staff: “Maybe examples…what contingency management is in a way where medical and clinical staff can understand it and where they can both implement it. Potential examples of what maybe financial and non-financial incentives that we could use and maybe some different statistics on how effective it is. Then, maybe, lastly, ways where we could get the funding for the actual incentives.”
	• Learning tools	Leader: “Many of the people I supervise are very visual, so if there’s anything they could read. Examples, like case studies, they learn a lot by them, and really good, concrete ways to implement it, like a how-to guide…” Staff: “…I think a workshop for this would be more beneficial in kind of providing examples and maybe like a role play stuff. I think that would be helpful. We don't too much of that.”
Performance feedback	• Feedback source	Staff: “I think it would be appropriate to have done during our regular scheduled supervision hours that we have with our supervisors cuz they know us in the sense that they know how our delivery is, the type of provider we are, what our strengths and weaknesses both are…” Staff: “Honestly, if the patients are the ones that we’re doing it for, to have an evaluation on their end would probably make the most sense to me, because they’re the ones that we’re supposed to be trying to affect. In my mind, that feels like the best option.”
	• Feedback delivery	Leader: “Interestingly, I’ve noticed that our patients are more [receptive] to having a person sit in on a session than have it audio recorded.” Staff: “I, personally, wouldn’t have any issues having my supervisor use that [session recordings] as a way to give me feedback. I would think that if it was okay with a patient that a supervisor would observe the session…You can see, for yourself, the effectiveness of what’s happening as opposed to just literally hearing it on tape...”
External facilitation	• Facilitation format	Leader: “…Maybe, some sort of website or online support would be helpful…I think having somebody I could email and say , ‘Hey, I gotta question for ya,’ or ‘What would you do in this situation?’ or something like that would help.” Staff: “Email is this and email is that. It's great…when we come together as people and we're face-to-face, it's a little different...I might feel comfortable saying things or I might get a facial expression from you—oh okay, I guess I'm doing something wrong or doing something great, and have the support. I just think that in person is the best way.”
	• Facilitation duration and frequency	Leader: “perhaps a monthly check-in or office-specific staff meeting and then possibly quarterly in-services agency wide…” Staff: “…even a file on the computer we could pull up in case we have questions, or even an outside source that we could call, so something like you…If you have questions, call this number.”

### Sample characteristics

Administrators from 11 of the 13 approached OTPs (85%) agreed to nominate staff. OTP leaders and directors nominated a total of 44 staff (22 leaders, 22 providers) to participate. Twenty-one leaders (95% of nominated) and 22 providers (100% of nominated) enrolled and completed qualitative interviews. Participants were primarily White (93%), female (72%), and had earned a bachelor’s degree as their highest education level (42%). Years of employment at their current OTP varied significantly among participants from 3.5 months to 41 years; average tenure was just under 5 years. There were no significant demographic differences between providers and leaders.

### Didactic training

Preferences regarding didactic training encompassed three emergent sub-themes: training format, content, and learning tools. In general, preferences for didactic training were similar for leaders and providers.

#### Training format

The training format sub-theme encompassed preferences pertaining to both the location and duration of training. Seven participants (4 leaders, 3 providers) shared their opinions about where didactic training should occur. Three leaders and two providers expressed a desire for on-site training at their OTP. One of these leaders noted the convenience of in-house training, stating that, “If somebody came out to us, that would be absolutely perfect.” By contrast, two participants (1 leader, 1 provider) explicitly stated a preference for off-site training; one leader emphasized the value of going to a secondary location for training by suggesting, “I think the workshops and seminars are good ‘cause it takes the staff away from here, and they can concentrate on just that.” Four participants made suggestions about training duration (1 leader, 3 providers). Preferences ranged from a minimum of “an hour” to a maximum of 2 days (“couple of days’ worth”).

#### Training content

With regard to training content, quotes repeatedly referenced a desire for case examples paired with research evidence. Requests for specific case examples of successful CM implementation were common (2 leaders, 4 providers). One leader suggested “…having concrete examples of where it’s been successful would be very helpful and how another agency may have implemented it*.*” Other respondents suggested that case examples would help with “buy-in” of OTP counselors and leaders.

Six participants (4 leaders, 2 providers) shared impressions about the value of objective research evidence as a teaching tool. For example, one leader stated, “Definitely giving them just a literature review… perhaps I’m old-school and very academic, for that’s the best way to disseminate information.” Remaining quotes supported the value of research data in convincing staff that “it’s not just someone’s idea” and inspiring them to adopt a new intervention.

#### Learning tools

In terms of learning tools, participant comments reflected a desire for active learning strategies during the training and supplementary resources to take home after the training. Two participants (1 leader, 1 provider) requested experiential strategies such as role plays and behavioral rehearsal, and both thought that active learning could help the staff become “comfortable” with the intervention. As an example, the leader recommended, “... doing a few role-plays sometimes helps for some people…I think just making sure they have all the tools in their tool belt, as we say, to make sure that they can implement it.”

Meanwhile, two participants (1 leader, 1 participant) spontaneously requested supplemental resources such as handouts and training materials as a means of helping training participants to retain information. The leader explicitly recommended handouts, noting “...[staff] love handouts, because if they’re not hearing you because they’re burnt out that day, they have somethin’ to take with them.” Similarly, the provider recommended that all didactic materials be compiled and shared after the training, noting, “when trainings happen, you’re not able to get all this information in one shot. You can try, but you can’t so if everything could be in a nice packet…”

### Performance feedback

In the performance feedback theme, sub-themes that emerged included the feedback source (i.e., who would provide performance feedback) and the feedback delivery (i.e., how feedback would be evaluated and shared with the provider).

#### Feedback source

Participants suggested several potential sources of performance feedback, with substantial consistency between providers and leaders. The most common recommendation (10 leaders, 17 providers) was an in-house supervisor. Participants suggesting this option generally cited a desire for comfort and rapport with the individual providing feedback. For example, one provider explained “I just think getting feedback from somebody you don’t know is a lot tougher than getting feedback from somebody you have supervision with once a month.”

The next most popular suggestion (5 leaders, 15 providers) was to receive performance feedback directly from CM patients. Respondents recommending this option shared the belief that the patients would have the most “accurate information” into how helpful the counselor’s CM delivery was for enhancing their normal care. Other providers advocated that patient feedback should be a central component of intervention evaluation, because patients are the target end user (i.e., “who I’m helping”).

Finally, seven participants (2 leaders*,* 5 providers) indicated a preference to receive performance feedback from a CM expert outside of their clinic. Various benefits cited of expert feedback included objective input, reduced potential for conflict between co-workers, and assurance of equitable feedback. For example, a provider shared her opinion that, “the best bet would be someone outside of here. It would become unequal, I think, if it was someone within the clinic.” Similarly, a leader shared the view that “somebody that doesn’t know us that well, or hasn’t worked with us, and doesn’t have a personal relationship, is gonna tell us the truth and is gonna lay it [feedback] out how it needs to be laid out.”

#### Feedback delivery

Regarding feedback delivery, providers and leaders shared their impressions about both audio recordings and rating scales. Of the thirteen participants that expressed their opinions on the use of audio recordings for feedback, five participants (3 leaders, 2 providers) were in favor and eight (3 leaders, 5 providers) expressed some concern over such a tool. Those in favor touted the potential for high quality feedback. For example, one leader shared, “…We have done that with MI [motivational interviewing] where we’ve had to tape ourselves with a patient and then send that out, and then get a ratings sheet on that, that could be a good idea.” By contrast, other participants expressed wariness over audio recordings as a viable option due to concerns about discomfort (both their own and their patient’s), as indicated in this provider’s response, “Yeah, it’s tough. As far as recorded, I, personally, don’t feel like any of my patients, or a very limited number of my patients, would feel comfortable having anything documented on record so openly such as that….”

Feedback about performance feedback rating scales was more consistently positive. A greater number of providers than leaders (5 providers, 3 leaders) shared positive feedback about such scales, with six recommending them in the context of supervision and two recommending them in the context of patient care. Beyond explicit suggestions of rating scales, participants also had positive views about performance evaluations using patient surveys/questionnaires (3 providers, 2 leaders) and CM checklists (2 providers). One leader shared her endorsement of scales, noting “With a checklist or with the rating scale, you can see it. Then when you’re talking about it, you can process through what’s getting in the way. I like rating scales.”

### External facilitation

Participants also reported on their preferences for external facilitation. Responses indicated interest in either a remote support system (2 leaders, 6 providers) or in-person contact (4 leaders, 3 providers) offered in-house. Nine leaders and providers (2 leaders, 7 providers) expressed a desire to receive “as needed,” “on-call,” or “as things happen” ongoing support. In addition, ten participants (6 leaders, 4 providers) expressed a desire for additional structured facilitation sessions at pre-scheduled intervals. The most popular suggestion for the frequency of sessions was monthly (4 leaders, 2 providers), closely followed by quarterly (2 leaders, 2 providers). Requests for support included help with both CM delivery (e.g., “type of incentive [CM] for someone, and …how we’re going to do it”) and with CM implementation (e.g., “track it how we’re as a staff buying into it”). Desired support for help with intervention delivery included a number of issues unique to CM implementation including how to monitor whether CM was working for patients, how to identify which patients earned prizes, and how to monitor and award the actual prizes. Meanwhile, ideal support for help with CM implementation encompassed topics such as promoting staff “buy in,” providing ongoing training via seminars and workshops, and having ongoing monitoring of staff CM use.

Remote facilitation recommendations encompassed a range of options including email, phone, or video-conference sessions. One provider shared her perspective that remote support would help her to feel more confident about intervention delivery: “If I could get the facilitator’s contact information to send them an email about something if I needed help in a situation—Just knowing that I have the support, I think I’d feel a lot better.” Meanwhile, those participants that advocated for in-person support noted the convenience of being able to consult with a facilitator in the course of routine operations. Some participants suggested having the facilitator drop into the OTP at random to check in, while others suggested having the facilitator join at pre-determined intervals (e.g., at routinely scheduled staff meetings).

## Discussion

This study conducted user-informed formative research (e.g., recruiting potential users, defining target users and their needs, and conducting interviews with target users to understand their preferences) to solicit feedback from OTP leaders and front-line providers about a comprehensive CM implementation strategy. Emergent themes analysis informed adaptation of the SSL implementation strategy for delivery to OTPs in a large hybrid 3 implementation-effectiveness trial (see Curran et al. for details on hybrid trial designs [26]). In general, there was high concordance between providers and leaders in terms of their preferences.

With regard to didactic training, respondents indicated a preference for a relatively brief (e.g., half-day to 2 days) workshop, buttressed by case examples, research data, experiential learning, and resources. These findings are in alignment with previous research regarding effective aspects of didactic training, as experiential learning strategies (i.e., role plays) have been shown to expand a workshop’s potential to increase intervention skills and subsequent implementation with patients [13, 14]. Role plays act as a form of behavioral rehearsal (i.e., rehearsal of how CM will be delivered with a patient), which increases training success and intervention fidelity [27]. Literature also suggests that the provision of case examples renders evidence-based interventions more compelling and increases clinician interest in gaining training [28]. Feedback from OTP staff was highly consistent with the SSL model, which typically consists of 1 day of didactic training pairing research data with experiential learning, and suggests that the inclusion of CM-focused case examples and resources would be of significant value to OTP staff [18].

Participants had varied views on how to best receive performance feedback but were generally in favor of receiving feedback to enhance CM fidelity, particularly in the form of objective rating scales. Some respondents preferred feedback from external CM experts, though more respondents were comfortable getting feedback from an internal source (i.e., a supervisor) or from their patients directly. These findings suggest that future research on our SSL approach, typically reliant on external technology transfer specialists, might benefit from evaluating additional CM training for internal clinical supervisors (i.e., a train-the-trainer approach [29]). A train-the-trainer model could enhance provider comfort with receiving performance feedback and improve CM sustainability potential by limiting the need for continued external support [30].

Respondents also had varying views of the utility of audio-recording CM sessions, with some highlighting the utility of recordings and others expressing concerns about patient privacy. Participants’ general receptivity to feedback was encouraging given the literature supporting the effectiveness of performance feedback for enhancing training outcomes and maximizing evidence-based practice fidelity [11, 12, 15, 17, 31]. The ambivalence about audio-recordings was not surprising given the brief tenure and limited education (i.e., bachelor’s level of education) of many OTP providers in the current sample [32]. Indeed, prior work has demonstrated that providers with limited training may experience increased evaluation anxiety when receiving supervision on audio or video-recorded sessions [32]. Though common, such ambivalence presents a unique training challenge given that audio-recordings are considered one of the gold-standard approaches for performance and fidelity monitoring [33, 34]. These results suggest that the SSL strategy would likely benefit from inclusion of an explicit orientation to the performance feedback process that clearly outlines expectations for the use of audio recording to socialize OTP staff into their role and expectations [32]. Additionally, to assure OTP staff of equitable, fair assessment, feedback would ideally be provided via well-validated scales such as the Contingency Management Competence Scale (CMCS [35]) to measure the quality of CM delivery.

In terms of external facilitation, participants expressed an interest in both on-call/as needed consultation and more structured remote support (ideally offered monthly) to help them learn CM skills and troubleshoot problems while implementing CM. Facilitation has been identified as a core component of novel practice implementation across numerous studies, many of which have used an external coach or facilitator to enhance the effectiveness of didactic training [31, 36]. The combination of formal and informal support is also a key component of the SSL implementation strategy: a technology transfer specialist offers partner sites formal monthly facilitation calls and informal consultation as needed, focused on addressing obstacles to implementation [18]. The current results suggest that for OTP staff, the facilitation sessions should not only focus on implementation support, but also on skillful delivery of CM, given some of the unique challenges associated with CM intervention delivery.

### Implications: user-informed modifications to the SSL implementation strategy

The current study suggested that the SSL three-tiered implementation strategy would benefit from adaptations to improve fit with OTP staff, many of whom had limited experience at their OTP, familiarity with CM, and higher education. Our research team made several key adaptations to each component of the strategy to balance OTP staff feedback while also maintaining the SSL’s key evidence-based components (didactic training, performance feedback, and external facilitation). First, we adapted our typical 1-day CM didactic workshop by reducing the amount of time spent on research data and increasing time spent discussing case examples documenting successful CM implementation (including review of behavioral targets, prizes, and reinforcement schedules) and engaging in experiential learning. We also augmented the workshop with a plethora of CM resources (including training videos and recorded role-plays), made highly accessible via a project website (https://www.sites.brown.edu/projectmimic).

Additionally, we added explicit training content orienting providers to the audio-recording process and required that providers submit an audio recorded role-play (rated as adequate on the CMCS) prior to CM delivery with patients. To address participants’ preference for performance feedback from an internal supervisor, we had each site identify 1–2 leaders who would be responsible for supervising CM delivery in the longer term. Identified leaders received monthly performance feedback reports on their providers’ CM delivery (i.e., copies of their CMCS performance reports) and CM implementation (i.e., consistency and reach of CM delivery). Identified leaders received instruction to use the CMCS in order to institutionalize performance feedback after removal of active support. This approach was a more feasible first step than a train-the-trainer model given the large number of partner programs and the need to monitor trainer fidelity; however, as noted earlier, evaluating train-the-trainer models is a worthy direction for future research.

Finally, we offered two distinct monthly remote facilitation sessions: one led by a national CM expert and another led by a technology transfer specialist to provide support in both intervention and implementation delivery. This was a significant change to the SSL approach as external facilitation is typically only provided by technology transfer specialists who are experts in implementation support, but not in the actual intervention. In between remote facilitation sessions, OTP staff could call a project hotline answered by multiple research staff with any questions about either CM delivery (e.g., how to calculate prize draws if a patient missed a session) or the nuts and bolts of implementation (e.g., how to use the audio recorder).

### Limitations

Several limitations may impact the implications of these findings as this work is a first step in obtaining user feedback about the SSL strategy. Our sample consisted of primarily White, female providers, which may have implications for whether these findings transfer to other populations or users of the SSL strategy [37]. Of note, these demographics are representative of addiction treatment providers in New England [38], highlighting a need to improve diversity in the workforce at large. Next, our sample consisted of providers nominated by their organization for participation. This recruitment method introduces the potential for selection bias by highlighting the perspectives of the strongest CM leaders and providers at their agencies. Finally, we acknowledge that participants’ verbalized preferences for CM implementation may be prone to social desirability (i.e., due to speaking with research staff who may be perceived as vested in the training). We attempted to mitigate against this concern by conducting this study with different OTPs than those that ultimately participated in the cluster randomized trial.

## Conclusions and future directions

These limitations notwithstanding, the current study represents a novel attempt to apply formative research to adapt a multi-level implementation strategy to improve fit within the OTP specific context. While this study did not engage in a comprehensive user-centered design approach (see Lyon et al., 2019 and Lyon & Koerner, 2016, for descriptions of comprehensive approaches [39, 40]), the use of interviews to inform SSL modifications for community-based OTPs represents a potential first step in adapting an implementation strategy in line with the Discover phase of the Discover, Design, Build, and Test (DDBT) user-centered design framework [39]. User-centered design principles provide an opportunity to develop and adapt both interventions and implementation strategies with the end user in mind through the use of stakeholder feedback [40, 41]. The Discover phase of the DDBT framework focuses on discovering areas for strategy modification by identifying influential stakeholders (i.e., OTP leaders and providers), evaluating stakeholders’ needs (i.e., organizational contextual factors), and identifying barriers or usability issues through interviews, focus groups, direct observation, and usability testing (i.e., direct interaction with SSL techniques [39]). Results of the current study highlighted a number of needs and barriers to the SSL that informed an initial set of SSL modifications including adjustments to each of the three elements of the evidence-based implementation strategy: didactic training (e.g., structure, format, techniques used), performance feedback (e.g., source and frequency of feedback), and external facilitation (e.g., source and frequency of facilitation). Future research could build upon the methods employed in the current study and apply user-centered design principles from the Design and Build phases of the DDBT framework to beta-test and refine implementation strategy elements prior to formal testing and deployment in a specific setting. The DDBT framework would also facilitate a process of determining the transferability of study findings to other populations and settings seeking to employ the SSL [39, 42].

Overall, the current study serves as a model for using a user-informed approach to modify existing implementation strategies to maximize their fit in novel settings. User-informed adaptation of implementation strategies is not often employed in the implementation science literature but has the potential to increase the uptake of both an implementation strategy and the evidence-based practice being implemented. In an ongoing cluster randomized trial, our team will specifically evaluate the extent to which the adapted SSL strategy is associated with improvements in both implementation outcomes (i.e., CM exposure, CM skill, CM sustainment) and patient outcomes (i.e., patient abstinence, patient attendance).

## Data Availability

The data that support the findings of this study are available on request from the corresponding author KS. The data are not publicly available due to them containing information that could compromise research participant consent.

## Abbreviations

MOUD: Medication for opioid use disorder
OUD: Opioid use disorder
CM: Contingency management
OTP: Opioid treatment program
SSL: The Science-to-Service Laboratory
ATTC: Addiction Technology Transfer Center
CMCS: Contingency Management Competence Scale
DDBT: Discover, Design, Build, and Test Framework
